# Meaningful higher education in Kakuma refugee camp: A case study of why context and contextualization matter

**DOI:** 10.1007/s11125-022-09610-z

**Published:** 2022-09-07

**Authors:** Paul O’Keeffe, Thibault Lovey

**Affiliations:** 1 UCD Global, University College Dublin, Dublin, Ireland; 2 Department of Public and Global Health, Division of Infectious Diseases, Epidemiology, Biostatistics and Prevention Institute, University of Zurich, Zurich, Switzerland

**Keywords:** Refugees, Blended learning, Higher education, Contextualization, Refugee camps, Technology

## Abstract

In recent years, higher education in refugee contexts has begun to receive increasing attention within the humanitarian-development sector. Resource constraints, coupled with the technology and innovation zeitgeist in international development drives, have helped to create a higher education space where courses in refugee camps are typically delivered via online learning platforms directly from Western education providers. As the space develops, a shift in attention is beginning to occur, such that the legitimacy of online learning for refugees is now being questioned. At the heart of this question are the issue of contextualization and a call for greater emphasis to be placed on blended learning approaches that better reflect the realities of refugee learners. In this case study, the authors compare and evaluate a contextualized medical studies course that was delivered via blended learning in the Kakuma refugee camp in 2019 with a non-contextualized version of the same course that was delivered in the Dadaab refugee camp in 2018. The study explores the contextualization process and finds that the contextualized course achieved better learning outcomes than did the non-contextualized version of the course.

Higher education programs in refugee camps are seldom created, administered, or delivered by refugees. Resource shortages; employment restrictions; capacity limitations; and various other social, political, geographical, and economic factors have resulted in a higher education space that is dominated by online learning imported via information communication technology (ICT) platforms and, to a lesser extent, via Western education providers’ “campus” models (Halkic & Arnold, 2019). The majority of courses that refugee learners participate in are rarely contextualized to include the particularities of refugee life, nor are they delivered through means that best reflect the pedagogical needs of refugee students (Crea, 2016).

Furthermore, relevance, meaning, and resources are often evaluated after the delivery of higher education courses for refugee learners (Akkari & O'Keeffe, 2021; Carron, 2019). As academic investigation into higher education in refugee contexts (HERC) is a relatively novel academic pursuit, with a limited body of literature available (Bellino, 2018; Taylor & Sidhu, 2012), it is difficult to assess the broad impact of HERC on refugee learners. However, the emerging research that is available supports the need for greater thought and investment into higher education programs that suit the specific needs of refugee learners, rather than importing courses not designed for these learners or the context in which they live. For example, a meta-analysis of research into HERC (Ramsay & Baker, 2019) found that it was enriching and rewarding for refugee students and could meet their distinct needs when it was rendered relevant and meaningful to those who participated in it.

The hegemony of the online learning model in HERC is closely linked to and facilitated by significant structural transformations taking place in the humanitarian field. From refugee registration being outsourced to the multi-million biometric industry to data and digital technologies proliferating in most United Nations agencies (primarily in the form of innovation labs and data departments), datafication and digitization, along with increasing privatization, have become ubiquitous within the humanitarian action sphere (Madianou, 2019). The HERC sector is no exception. In conjunction with this, policy drives within the international forced-migration management system for increasing access have ushered in an era that aims to leverage technology to increase educational opportunities for a growing number of refugees worldwide (Colucci et al., 2017; Dahya, 2016; Miao et al., 2018; Traeger & Löwe, 2018).

The growing emphasis on the role technology can play in HERC is not without its detractors. Chief amongst these are academic researchers concerned with the continuities of global inequalities, as typified by the imperial formations that frame the lives of displaced people around the world (Madianou, 2019; Stoler, 2016). For example, critics often point to a fixation on “innovation” in this space as justifying experimentation with refugees (Sandvik et al., 2017), which would not be tolerated by affluent people in Western countries (Magalhães, 2021; Mann, 2018). This emerging interrogation of what has become known as *technocolonialism* (Madianou, 2019) is closely bound up with the longstanding decolonizing education debate, which calls for space to be made for Indigenous thought systems within the dominant Western episteme, methodologies, and scholarship (Hendricks & Leibowitz, 2016). In the HERC sector, a growing shift in focus from access and implementation to inclusion and, more pertinently, contextualization of curricula has opened up a space where the parachuting of Western-centric higher education approaches and thought into refugee contexts is starting to be examined.

It is well established that contextualization of course content and concepts improves students’ motivation, learning, and persistence (Krause et al., 2016). Contextualized education involves linking foundational skills with academic content by focusing teaching and learning on concrete applications in a specific context that is of interest to the student (Mazzeo et al., 2003). In short, it is significant, it is meaningful, and it is relevant to the student and asserts that they will more easily assimilate knowledge they can more readily understand (Andriotis, 2017).

The seminal book *How People Learn* (Bransford et al., 1999) laid out the cognitive processes that act to achieve learning through conceptual change and provided a framework from which contextualization of content can be understood. The authors pointed to the following three principles that aid effective learning: (a) identifying prior knowledge to inform instruction, (b) engaging students to promote conceptual change in order to construct deep knowledge, and (c) encouraging metacognition in students by allowing them to define their own learning goals and monitor their own progress (Krause et al., 2016).

The relevance of contextualization has been accepted in Western academia, and its importance permeates the literature (Wyatt, 2015). Grounded in constructivist learning theory, this acceptance of contextualization acknowledges that people learn better when encouraged to link instructions with their interpretations of these instructions within the context of their own environments. Mainstream pedagogical approaches, such as those that follow motivation and social theories and problem-based learning, draw deeply on the application of contextualization in their practice (Andriotis, 2017). From the traditional face-to-face to blended and online courses, the contextualization of aims, objectives, and outcomes frequently inform course design, implementation, and evaluation.

As mentioned, the internationalization of education through Western perspectives (Majee & Ress, 2018) and the datafication and digitization zeitgeist in the international forced-migration management system have enabled a space where online learning is de rigueur in HERC (Halkic & Arnold, 2019). Its position as the primary learning solution for refugees is further compounded by the various barriers that impede face-to-face and, to a lesser extent, blended learning approaches. The bedrock of online learning in HERC are massive open online courses (MOOCs), which provided the necessary technical equipment is in place, can be accessed at the click of a mouse. Even when technology and other resources are adequate, MOOCs have notoriously high dropout rates. In a synthesis of literature looking at online retention rates, Bawa (2016) found that between 40% and 80% of online students dropped out of online courses. In refugee contexts, where basic needs such as food and shelter are frequently not met and internet access and electricity are the exception and not the rule, online learning approaches that do not consider the context in which the learners live are suboptimal (Carron, 2019).

Furthermore, while the contextualization of MOOCs for specific audiences may be counterintuitive, they are generally built with a specific demographic profile in mind: well-educated Western professionals (Anders, 2015). Relevant contextualization of MOOC-based courses can only happen if blended or face-to-face approaches are taken, whereby the MOOC content and the teaching approach are adapted to the context of the students and are responsive to their needs (Fortus & Krajcik, 2012; Roseman et al., 2008).

In the realm of HERC, making content meaningful and relevant through contextualization and adapting teaching practices to meet the needs of refugee learners become more important when the specific learning needs of refugee learners are considered. Beyond the obvious needs (e.g., electricity, technology, internet connections, and adequate sustenance), refugee learners are people who often require additional educational care to help them cope with the unique challenges they have faced to get where they are. The neurodevelopmental effects of trauma, separation from extended families, broken community ties, disrupted education histories, culture shock. and so on all impact heavily on the educational performance of refugee learners (Schleicher & McLaughlin, 2018). Education can have a protective effect and provide the additional support needed for refugees when it is of good quality, maintains motivation, and encourages resilience (Miao et al., 2018). The pedagogical support permeating relevant and meaningful content and contextualized teaching approaches provides this critical protection and drives refugee learners to navigate beyond the social and emotional educational barriers unique to their situation (Brenner & Kia-Keating, 2016).

Putting the social, emotional, and psychological support that contextualization provides for refugee learners to one side, HERC courses are ultimately evaluated on the perceived impact they produce. Impact studies and their data-driven bottom lines are, for better or worse, the oil that fuels the majority of humanitarian projects, HERC included, by justifying aid budgets through the logic of accountability and audit that controls humanitarian organizations and their expenditures (Magalhães, 2021).

Evaluating contextualization of curricula and its impact in the wider HERC sphere presents many unquantifiable prospects, and thus it may not be easy to justify its importance over providing access to higher education courses on a mass scale. With this in mind, we lay out the following case study of a contextualized blended learning basic medical training higher education course in the Kakuma refugee camp, Kenya, and compare it with a non-contextualized version of the same course that had previously been delivered in the Dadaab refugee camp. By doing so, we aim to elaborate on the contextualization process and evaluate whether or not contextualization of content and pedagogical delivery had a positive impact on learning outcomes for refugee learners who took part in the Kakuma course, and thus justified the extra input required to contextualize the curricula.

## Case study

### InZone-Raft basic medical training course

**Figure 1:**
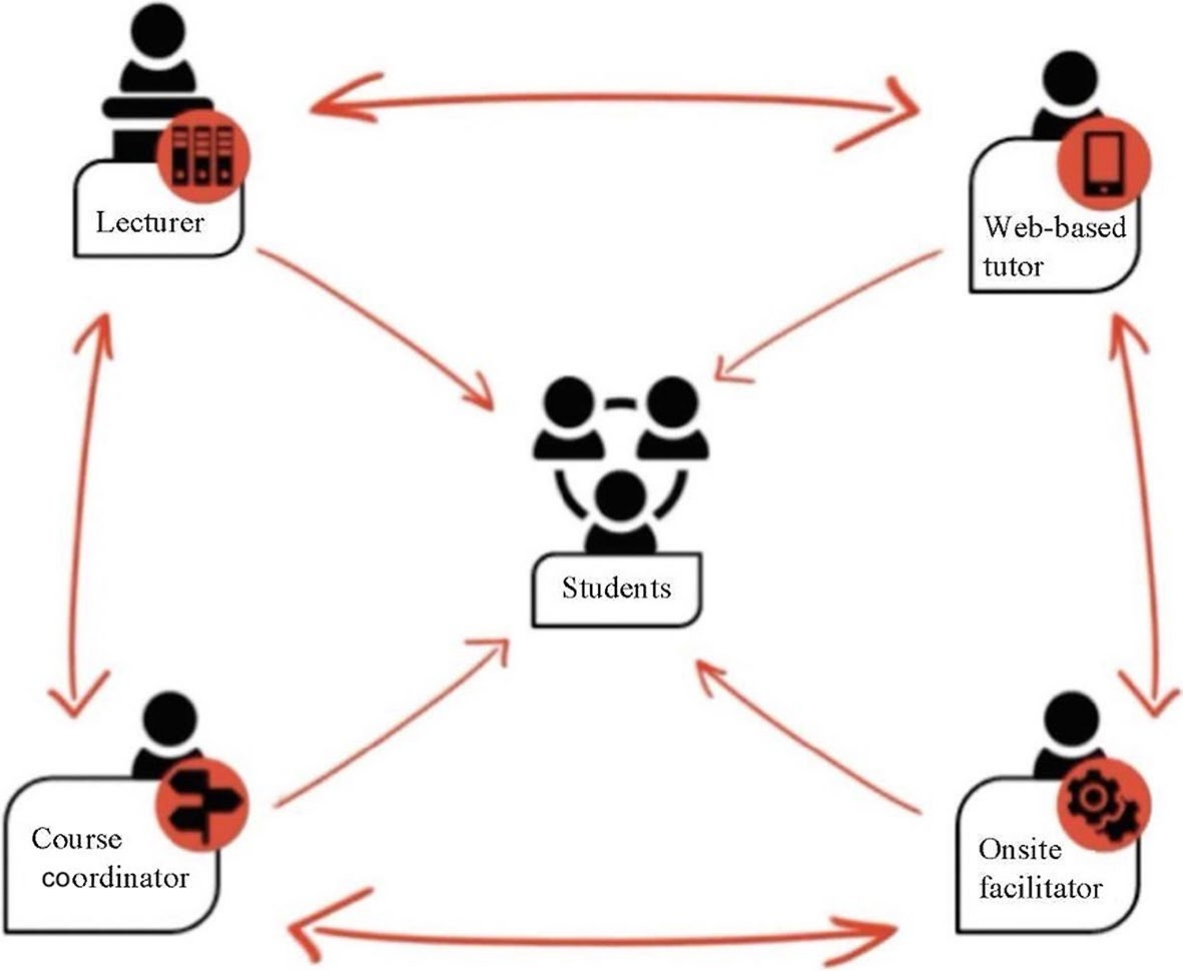
The InZone collaborative learning ecosystem

The InZone-Raft basic medical training course was a project of the University of Geneva that was delivered in the Kakuma refugee camp between 2017 and 2020. InZone is an academic and humanitarian program that operates in the Azraq (Jordan) and Kakuma (Kenya) refugee camps, using a refugee-led management approach in which refugees oversaw course implementation and contributed to course development and delivery. The program used a collaborative blended learning ecosystem to bring refugee students, web-based tutors, onsite facilitators, lecturers, and course coordinators together to enable accredited blended learning courses from the University of Geneva in the refugee camps. InZone has a center, (the learning hub) in Kakuma, where students congregate to access computers and Wi-Fi and connect to online courses from the University of Geneva. Learning materials are delivered via the University of Geneva’s online learning platform and supported by web-based tutors (typically graduate students at the University of Geneva) and trained refugee onsite facilitators, who manage the daily learning in traditional classroom settings on the ground. The InZone collaborative learning ecosystem heeds the context by scaffolding learning with pastoral and pedagogical support from the tutors and facilitators and by taking a student-centered approach wherein learning materials and lectures are adapted to meet students’ needs and better reflect the realities of their daily lives. Typically, the learners participate in online tutorials with their tutors via WhatsApp instant messaging, for a designated amount of time each week throughout a course, to support their acquisition of knowledge through the online learning platform. The onsite facilitators provide an extra layer of support and education protection by assisting the tutors and students in managing the classes and the learning process. Figure 1 illustrates the role of each actor in the collaborative learning ecosystem; a detailed explanation of their function is provided. Funding for the program was provided by the University of Geneva.

### Key actor roles and responsibilities in the collaborative learning ecosystem

1. The lecturer delivers the course material over the online learning platform, encourages the generation of new knowledge, and evaluates the students’ learning. In the ecosystem, the delivery of knowledge via an online platform enables the transmission of information to the students, who through discussions with their tutors and colleagues, group work, and so on acquire and develop new knowledge.
2. The web-based tutor is a subject-matter expert or a peer with a more advanced level of subject knowledge. The tutor plays a pedagogical role in the collaborative learning ecosystem by meeting the students regularly over an ICT platform (e.g., WhatsApp) to stimulate new knowledge acquisition, discuss the students’ progress, and offer advice on being a successful learner. The tutor also travels to the camp to meet the students in person and deliver face-to-face classes toward the end of the course.
3. The onsite facilitator provides onsite technical support and guidance to learners, helping them to access the learning platform on location and navigate the physical learning space. The onsite facilitator is a critical contact point in the educational relationship between the students and the other members of the collaborative learning ecosystem, as they are in frequent physical contact with the students. In conjunction with the web-based tutor, the facilitator encourages the students to gradually become independent learners.
4. The course coordinator has the overall responsibility for the day-to-day running of the course and liaises with the other members of the learning ecosystem to ensure a smooth operation.
5. The students are the focal point of the collaborative learning ecosystem. This means that they are central to the collaborative learning model, and the entire learning ecosystem is designed to support their optimal learning by meeting their educational needs and promoting progressive learner autonomy.

### The contextualizing process: From design to evaluation

In 2017, following the successful implementation of the InZone-Raft basic medical training course in the Dadaab refugee camp in Northern Kenya, InZone was approached by the United Nations High Commissioner for Refugees (UNHCR) and asked to adapt the course for refugees living in the Kakuma refugee camp. To do this, InZone partnered with the Raft Telemedicine Network at the University of Geneva and created a version of the course that could be enabled through InZone’s collaborative learning ecosystem in the camp. Although Dadaab and Kakuma are different places, with different population profiles (Dadaab is primarily populated by Somali refugees, whereas Kakuma has refugees from more than 24 different backgrounds), both camps are similar in terms of the poor conditions in which people live and the pedagogical challenges they face. Through lessons learned in the Dadaab course (Burkardt et al., 2019), the curriculum was contextualized to better meet the needs of refugee learners, and the pedagogical methodology was enhanced to accommodate the conditions and context of the Kakuma refugee camp (Lovey et al., 2021). Out of a pool of 50 applicants, 16 were chosen according to their preexisting educational qualifications, knowledge of health care, and motivation to take part in the course.

The course consisted of three modules. Module 1’s content covered an overview of the human body’s main organ systems; specifically, it covered the physiology and anatomy of 13 organ systems. Module 2’s content covered basic illness physiopathology specific to health issues encountered in sub-Saharan Africa. Module 3 was a case-based learning unit in which students examined nine patients’ medical problems. This case study focuses on the implementation of Module 1 of the course and explores how lessons learned during the first version of Module 1 in Dadaab were taken on board to inform the contextualization process for the design and implementation of Module 1 in Kakuma.

### Course design

The first lesson learned from the Dadaab experience was that an effort needed to be made to align the design of the course more closely to the needs of refugee students. Module 1’s extensive content covered 13 complex organ systems and was delivered over a limited period of time. The Dadaab course content was delivered using the *OpenStax Anatomy and Physiology* e-book. Post-course delivery feedback from the Dadaab students revealed that they found the materials used in the course to be stodgy and too complex. To mitigate these concerns for the Kakuma version of the course and create more dynamic content, videos from Khan Academy, which provided expert content, were included in the curriculum. With a deconstructed approach that simulated an interactive course and used popularized content, the intention of adding these videos was to make it easier for students to learn by increasing their motivation and satisfaction. Because the physiology book was the primary reference source for the Dadaab students, it was repurposed as an additional reference to the Khan Academy content for the Kakuma course.

In an effort to mitigate the problem of content being too complex, a detailed procedure was developed for each unit of Module 1 to better guide students through the learning process. This procedure involved introducing learning outcomes and objectives for each unit (which the students had to master for their final evaluation). The content was presented in a sequential logical manner using the Khan Academy videos and book chapters related to the subjects studied. Where relevant, additional resources were added to help students understand difficult concepts in the unit.

To monitor the students’ knowledge acquisition and assist their efforts to become independent learners, quizzes were administered at the end of each unit. This quiz format had already been used in the Dadaab course and found to be helpful to the students, so it was decided to integrate quizzes into the new procedure. Students could choose between three quiz levels of difficulty (easy, medium, hard). Each level contained 20 questions and could be repeated as many times as students felt necessary. The quizzes were designed to target the main difficulties of each unit, while staying aligned with the list of key concepts.

Tutoring is an integral part of the design of all InZone’s courses (O’Keeffe, 2020). As outlined, the web-based tutors played the main pedagogical role in the InZone collaborative learning ecosystem. Feedback from the Dadaab course revealed that the students placed great importance on the tutors’ input into their course. It was therefore decided to enhance the role of the tutor for the Kakuma course design and to use a more structured tutoring format in the course design. This involved the tutors receiving extensive context training from InZone on the organization of the camp, the status and living conditions of refugees, the climate, security issues, health care, and other issues related to life in the camp. The training also focused on communicating with refugee learners and on how to behave in case of a hostage situation or life-threatening incident when visiting the camps. The tutors used their newly gained contextual knowledge to update course content and tutorial delivery procedures. For example, each week, the tutors set a contextually relevant question for the students to discuss in the tutorials, based on their knowledge of life in the Kakuma refugee camp.

### Accessibility

The second lesson learned from the Dadaab course concerned improving accessibility for the learners. A refugee camp’s volatile environment and remote location can create certain limitations that influence students’ access to knowledge. Some limitations are always present, and others can be influenced by external factors, such as weather or camp dynamics.

With an average of 4 hours of electricity per day, internet access was more the exception than the rule in both the Dadaab and Kakuma refugee camps (Lehne et al., 2016). During the Dadaab course, computers and free Wi-Fi were made available to the students. In addition, flash drives preloaded with course materials were available onsite in case of power outages or internet connection issues. Some students directly connected the preloaded USB sticks with a USB OTG adapter to their smartphone so they could move around the learning facilities and interact with their classmate while having the course in front of them. While this effort improved accessibility to knowledge for students when they were present in the learning facilities, it did not improve accessibility for students who did not have a smartphone and could not access the learning facilities. To mitigate this barrier for the Kakuma students, a Huawei Media Pad T3 10 tablet with the preloaded course materials was distributed to each student instead of the USB sticks. This allowed them to study from their shelters during times when access to the learning facilities was not possible and/or internet access was not available.

Both refugee camps are organized into separate sections where specific communities have settled over the years. As a result, some students had to walk long distances to reach the learning facilities, sometimes for several hours in extreme weather conditions (temperatures can easily reach 38C). In addition, as per course attendance requirements, students were required to attend lessons several times a week, meaning frequent trips to the learning facilities. Motorbike taxi transport costs to and from the learning facilities were paid to the students to ensure that difficulty moving around the camps was taken into consideration when managing their access to the facilities.

A final area related to contextualizing accessibility into the course was security. Following the do-no-harm principle (Anderson, 1999), efforts were made to ensure that classes and trips to the learning facilities were cancelled if any potential risks to the well-being of students were perceived. For example, in the event of an unexpected flood that would make traffic in the camp impossible, students were asked to stay at home. In such situations, tutorials were postponed or deadlines were extended to accommodate any unforeseen disruptions to the students’ schedules.

### Communication

The final lesson learned from the Dadaab experience, in relation to contextualization, was the need for appropriate communication within the infrastructure of the course. In line with the collaborative learning ecosystem model, a refugee was designated as the onsite facilitator for the course. As discussed, the facilitator’s main function was to provide technical support and guidance to the learners. This privileged access to the students allows rapid communication with course leaders located in Geneva, who could make adjustments to the course if difficulties were encountered with content or from external events. This pivotal point was central to the Dadaab course and was continued in the second version of the course in Kakuma.

In the Dadaab course, the online communication platform WhatsApp was used as the main communication tool to interact with students (it is free and widely used in both camps). Through it, a group was opened for students to freely communicate their questions with their classmates and tutors. A weekly structured 2-hour tutorial was organized at the beginning of each week on the platform. In it, students’ questions were answered, and engaging questions were put forward by the tutors. The WhatsApp discussion group format was carried over to the Kakuma course and, as elaborated on already, was further used in the Kakuma course by the tutors to lead discussions on contextually relevant learning points each week.

During the Dadaab course, a major difficulty encountered was encouraging the students to be proactive in their studies. In response, individual tutorials were implemented, which yielded little success. However, group work approaches were well received by the refugees, which indicated their need to come together at the learning facility. With this in mind, more effort was made to encourage students to share their questions and difficulties with their colleagues in the Kakuma course. Self-organization, as a key component of independent learning, was encouraged by the tutors, who worked closely with the facilitator to guide the students. This was done by the facilitator, who helped the students prepare their own reading materials on difficult topics and arranged for them to meet up as a group to share their resources.

### Evaluating contextualization

Quantifying contextualization and its impact on learning is not a straightforward task. To do so in detail goes beyond the scope of this study. Our evaluation of whether or not contextualization improved learning for the basic medical training course relied on comparing course attendance rates, collaborative interactions, and the results achieved by the Kakuma students with those achieved by the Dadaab students. Within the design of the course, in both locations, two exams were implemented and supervised directly by the tutors at the learning facilities when visiting the camps at the end of each course. These were a written exam (combining multiple-choice and essay questions) and an oral exam (including questions on two randomly selected learning points). The only difference in the exam procedure was the addition of questions to the oral exam in Kakuma to test students’ ability to use knowledge learned in a different context and apply it to a clinical case. These questions, while not taken into account for the final exam results, were added because it was found that the Dadaab students had not internalized knowledge well and relied mainly on memorization of learning points in their oral exam; thus, the effort was made to test if the Kakuma students had internalized this knowledge.

The following results section lays out the comparisons between the two cohorts in terms of course completion rates, exam results, interaction and participation via unit quizzes and the WhatsApp forums, and the feedback given by the students’ course evaluations. The results were calculated and provided by the tutors of each course.

## Results

Initially, 27 students were recruited to take part in the Dadaab course, and 16 were recruited to take part in the subsequent course in Kakuma. In Dadaab, 18 students completed the course and passed the final exam, whereas 11 completed the course and passed the final exam in Kakuma. Feedback collected from the participants in both cohorts revealed that the relative frequency of dropouts was 9/27 (33%) in Dadaab and 5/16 (31%) Kakuma. It was found that their reasons given for dropping out were almost identical in both locations. In Kakuma, family problems was the main reason given, followed by leaving the refugee camp, attending an alternative training course, and lack of commitment due to prospects of relocation. In Dadaab, the reasons given for dropping out were divided between moving to another camp, returning to the country of origin, and family problems (Burkhardt et al., 2019).

Evaluating both courses, Lovey et al. (2021) found a significant difference between them. Specifically, a significant difference was observed when comparing written exam result means in both groups, 30% and 51% (38/75), respectively (two-tailed test: *P* = .006 and one-tailed: *P* = .003) and when comparing oral exam result means in the Dadaab and the Kakuma course, 51% and 67% (13/20), respectively (two-tailed test: *P* = .05 and one-tailed: *P* = .03). The critical thinking questions that were implemented only in Kakuma showed an average of 50% (4/8) correct answers on a new clinical case, with one student getting a perfect result and another only 13% (1/8) of the total score (Table 1).

**Table 1:** Comparison between Kakuma and Dadaab cohort

	*n*	Mean score (SD)	Percentage	Range	*P*-value^c^
Kakuma group	11				
Written exam (total possible score: 75 points)		38 (14)	51	17–80	0.006
Oral exam (total possible score: 20 points)		13 (5.5)	67	10–100	0.05
Final results		—^a^ (21.9)	59^b^	14–88	0.008
Critical thinking (total possible score 8 points)		4 (2.6)	53	13–100	N/A
Quiz results (total possible score: 10 points)		5 (1.3)	48	29–69	N/A
Dadaab group	18				
Written exam		—^a^	30	0–56	N/A
Oral exam		—^a^	51	30–93	N/A
Final results		—^a^	41	15–71	N/A

^a^ Not available

^b^ Calculated by combining written and oral examination results weighted by half

^c^ Student *t-*test, Wilcoxon signed-rank test

N/A: Not applicable

Twenty-five quiz questions were reused from the Dadaab course in the subsequent Kakuma course. Among them, six questions were significatively different in their score: two in favor of the Dadaab course model and four for the Kakuma course model. The participation rate in the quizzes was higher among the Kakuma students, with a median of 91% (10/11). In Kakuma, students’ quiz scores were proportional to their written scores (ρ = 0.93; *P* < .001) and final score (ρ = 0.94; *P* < .001; Table 1)

The average number of messages per day on the WhatsApp forum was seven for the Dadaab course and four for the Kakuma course. Among the Kakuma students, the preferred day for messaging was Saturday and the preferred time was 11 a.m. No association between the activities on the WhatsApp forum and the different evaluation modalities could be observed in either course. Nevertheless, by examining Kakuma’s WhatsApp chat and classifying the words into 10 categories of feelings (anger, anticipation, disgust, fear, joy, negative, positive, sadness, surprise, trust) to assess sentiments, an analysis showed that positive feelings were the most representative.

Feedback collected from the Dadaab students on the learning tools used in their course revealed a preference for the textbook (12/13, 92%), their phones (7/13, 54%), and the WhatsApp group forum (5/13, 38%). This was in contrast with the Kakuma students, who used the Khan Academy learning videos as their main source of study (11/11, 100%), followed by the WhatsApp group forum (4/11, 36%) and only rarely the reference manual (2/11, 18%).

Module 1 was judged *too difficult* (9/13, 69%), *too dense* (11/13, 85%), and the time allowed to cover the topics studied *too short* (8/13, 62%) by students in Dadaab. In Kakuma, the results were more positive, with students rating the level of content (8/11, 73%), the amount of information to be learned (8/11, 73%), and the level of English (9/11, 82%) as *adequate*. The lack of teaching support, which was observed as a *major obstacle* (8/13, 62%) in Dadaab, was *never* (6/11, 55%) considered an obstacle in Kakuma. Only the time available to study fit the previous pattern, as it was considered *short* (7/11, 64%). The reasons mentioned by the Dadaab students for time being too short were family obligations, household chores, and work, while in Kakuma, the specifics were not listed.

The main challenges were the same for both cohorts. Internet connectivity and access to electricity (only 4 hours per day in Dadaab) were replicated in Kakuma, with issues related to electricity access (8/11, 73%) *every day* and internet access (7/11, 64%) *every day*. Similarly, access/transportation to the learning facility (6/13, 46%) was listed as the main obstacle for students in Dadaab, as it was in Kakuma (6/11, 55%). This was a matter of great concern because the Kakuma students accessed the learning facilities five times per week (median).

Finally, although women did not express more difficulties than men did in the courses, and a difference in scores between women and men was not significant in the Dadaab course, the Kakuma course evaluation revealed that, on average, women scored 40% lower than their male counterparts.

## Discussion

Ultimately, the success of any higher education course is dependent on how much knowledge its students gain throughout their studies. For better or worse, this is usually measured by the grades the students achieve in their final examination. In the results section, we saw that a higher percentage of students in Kakuma passed their final written and oral exams and the Kakuma students achieved, on average, better scores, compared with the Dadaab students. While it may be impossible to say definitively, the fact that more students passed their final exam and gained higher results in the contextualized course suggests that contextualizing led to better outcomes.

The improved contextualization and the addition of videos to the syllabus of Module 1 did not affect the drop-out rate but appears to have had an impact on overall student satisfaction. Students felt that the level of content and amount of information to be learned were adequate, from which we conclude that the change from using a book to using interactive videos changed the students’ overall approach to Module 1. Memorization of key concepts in the course also appeared to be higher, as evidenced by the higher scores of Kakuma students on identical quiz questions. Finally, the answers given to the critical-thinking questions the Kakuma cohort received revealed that some students, especially those with high scores, were able to use their newly acquired knowledge in a new clinical context. This gives the impression that the deconstructed learning system using video enabled the students to understand what they were learning instead of just memorizing it, and thus allowed the students to use their new knowledge outside the classroom.

Going deeper, the results elaborate on pedagogical interactions in the course (WhatsApp usage) and found that students’ knowledge acquisition was not determined by these interactions, even if WhatsApp remained the main source of study for the students. These tools of communication further enhanced tutoring sessions, student-tutor exchanges, and planning, while reinforcing classroom dynamics and supporting distance learning.

The results also show that the perennial problems related to access (e.g., electricity, internet connection, and transport to and from the learning facilities) affected both cohorts. While access issues affected all students, empirically access affected female students more. Under representation, cultural norms, the burden of childcare and other responsibilities, poor sanitary facilities, security, and other issues conspired to make access to higher education for female refugees particularly difficult. The lower grade attainment of female students, compared with that of their male counterparts, in Kakuma reflect this difficulty and needs to be addressed if a commitment to better contextualization is to be achieved.

As mentioned in the introduction, available literature on contextualizing learning materials for higher education in refugee contexts is sparse. The findings of this research support authors, such as Ramsay and Baker (2019), who claim that contextualizing materials for refugees adds meaning and value to their learning. The success of the students in Kakuma also supports Krause et al.’s (2016) assertion that contextualization of course content and concepts improves students’ motivation, learning, and persistence.

## Conclusions

The results of this comparative study support the idea that contextualization of content and pedagogy works well and provides better learning outcomes for students. On a practical level, the extra effort put into contextualizing this course added value to the course for the students who took part in it, and the associated research added to the growing evidence that higher education for refugees can and should be relevant and meaningful for refugee learners. In conclusion, we wish to promote the Bransford et al.’s (1999) principles for understanding the process of contextualization of content, as mentioned in the introduction, to offer a clear framework from which education professionals working in HERC can approach the contextual needs of their students.

Principle 1, identifying prior knowledge to inform instruction, is a baseline that most educational courses follow when admitting students. In the case of HERC, evidence of prior learning is not always readily available. Refugees often flee their countries without time to gather certificates of achievements and are seldom able or willing to approach their national authorities to obtain such documentation. Despite this difficulty, education providers have other options available, such as course entry tests, needs assessments of capability, and bridging courses that can ensure the compacity to succeed is achieved.

Principle 2, engaging students to promote conceptual change in order to construct deep knowledge, can be achieved by adapting pedagogical approaches to include role plays; incorporating student-led instruction (as is done with the incorporation of refugee facilitators in the InZone model) and relevant problem-based learning; and most importantly, using content feedback and feedforward in continuous course development.

Principle 3, encouraging metacognition in students by allowing them to define their own learning goals and monitor their own progress, is central to learning outcomes that strive to foster a culture of independent learning. By guiding refugee learners to manage their own learning expectations and apply their new knowledge to their own particular contexts, instructors can help to ensure that refugee learners become central to their own learning experience.

By following these simple principles to assist in the process of contextualizing learning materials and approaches, education providers working in HERCs can address many of the criticisms being leveled against the sector at the moment. Ultimately, as we have seen from this study, contextualization can lead to better outcomes for refugee students and therefore needs to be the driving force for the future of higher education for refugees.
